# Supplementary material for: Exploring the Association of Burning Mouth Syndrome with Depressive and Anxiety Disorders in Middle-Aged and Older Adults: A Systematic Review
Source: J Pers Med. 2023 Jun 19;13(6):1014. doi: 10.3390/jpm13061014 (PMC10305447; doi:10.3390/jpm13061014)
Supplement: Supplementary file 1 [file jpm-13-01014-s001.zip › jpm-2396317-supplementary.pdf]

Table S1. Search strategy used in the US National Library of Medicine (PubMed) and Medical Literature Analysis and Retrieval System Online (MEDLINE) and adapted to the other sources, according to selected descriptors.

Strategy	Descriptors used
# 1	(elders[tiab]) OR (older adults[tiab]) OR (elderly[tiab]) OR (oldest old[tiab]) OR (youngest old[tiab]) OR (middle-aged[tiab]) OR (mid age[tiab]) OR (middle age[tiab]) OR (middle aged[tiab])
# 2	(burning mouth syndrome[tiab]) OR (BMS[tiab]) OR (glossalgia[tiab])
# 3	(emotional stress[tiab]) OR (depression[tiab]) OR (anxiety[tiab]) OR (suicidal behavior[tiab]) OR (suicidal ideation[tiab])
# 4	(review[tiab]) OR (systematic review[tiab]) OR (narrative review[tiab]) OR (meta-analysis[tiab]) OR (editorial[tiab]) OR (letter[tiab]) OR (perspective[tiab]) OR (commentary[tiab])
	#1 AND #2 AND #3 NOT #4
Updated to: April 2, 2023	
